# Hydroxysafflor yellow A alleviates myocardial ischemia/reperfusion in hyperlipidemic animals through the suppression of TLR4 signaling

**DOI:** 10.1038/srep35319

**Published:** 2016-10-12

**Authors:** Dan Han, Jie Wei, Rui Zhang, Wenhuan Ma, Chen Shen, Yidong Feng, Nian Xia, Dan Xu, Dongcheng Cai, Yunman Li, Weirong Fang

**Affiliations:** 1 State Key Laboratory of Natural Medicines, Department of Physiology, China Pharmaceutical University, Nanjing 210009, P. R. China

## Abstract

Hyperlipidemia aggravates myocardial ischemia/reperfusion (MI/R) injury through stimulating excessive inflammatory response. Therefore, blockade of inflammatory signal is a potential therapeutic management for MI/R complicated with hyperlipidemia. Hydroxysafflor yellow A (HSYA, a monomer extracted from *Carthamus tinctorius* L.), was studied in this article to address that the regulation of inflammatory signal would alleviate MI/R combined with hyperlipidemia injury. High-fat diet induced hyperlipidemia worsened MI/R mediated heart injury (elevation of infarct size, CK-MB and LDH activity), activated TLR4 over-expression in hearts, released inflammatory cytokines (LPS, TNF-α and IL-1β) excessively. HSYA administration suppressed the over-expression of TLR4 and alleviated heart damage caused by MI/R complicated with hyperlipidemia. Furthermore, HSYA had little influence on MI/R injury in TLR4-knockout mice, which indicated that HSYA protected MI/R through TLR4 inhibition. *In vitro*, hypoxia/reoxygenation (H/R) coexisting with LPS model in neonatal rat ventricular myocytes (NRVMs) induced serious damage compared with H/R injury to NRVMs. HSYA decreased excessive secretion of inflammatory cytokines, down-regulated over-expression of TLR4 and NF-κB in H/R + LPS injured NRVMs. In conclusion, HSYA alleviated myocardial inflammatory injury through suppressing TLR4, offering an alternative medication for MI/R associated with hyperlipidemia.

Myocardial infarction (MI) is a leading cause of death and a major health problem worldwide^1^. Reperfusion of myocardial ischemia is an essential strategy to salvage tissue from inevitable death. However, restoration of blood flow after a period of ischemia tends to cause extra damage which is commonly referred to as ischemia-reperfusion (I/R) injury^2^,^3^. Hyperlipidemia is one of metabolic syndromes characterized by the high level of plasma cholesterol and presence of hyperlipidemia directly correlates with the risk of developing myocardial infarction^4^, which demonstrates that part of hyperlipidemic patients are complicated with myocardial infarction. The possible mechanism associated with the incidence of myocardial infarction in hyperlipidemia patients is inflammatory response, which is considered to be the pivotal cause of MI/R-induced tissue injury^2^,^5^. Toll-like receptor 4 (TLR4), one of the pattern recognition receptors, plays a crucial role in the induction of the inflammatory response and activates downstream signaling (such as nuclear factor kappa B (NF-κB)) in myocardial ischemia^6^,^7^,^8^. Activation of TLR4 induces the expression of NF-κB dependent proinflammatory cytokines, such as tumor necrosis factor-α (TNF-α) and interleukin 1β (IL-1β)^9^. It is reported that high-fat diet (HFD) leads to hyperlipidemia, and increases the concentration of serum lipopolysaccharide (LPS), which is a stimulator of TLR4^10^,^11^. Therefore, blockade of excessive TLR4 expression is an available therapeutic management for MI/R superimposed on hyperlipidemia.

Hydroxysafflor yellow A (HSYA, the chemical structure is shown in Fig. 1) is the main bioactive compound of a traditional Chinese medicine named *Carthamus tinctorius* L. Previous studies demonstrated that HSYA possessed various kinds of bio-activities, including anti-oxidation, anti-inflammatory actions, anti-platelet aggregation, anti-tumor and anti-myocardial injury effects^12^,^13^,^14^. It was reported that HSYA attenuated inflammatory response in ischemic stroke and LPS-induced acute lung injury via TLR4-dependent signaling pathway^15^,^16^. However, the effects of HSYA on MI/R overlying hyperlipidemia and the possible mechanism are still unknown.

Hence, in the current study, we investigated whether HSYA mitigated MI/R superimposed on hyperlipidemia injury and the role of TLR4 in this process.

## Results

### HSYA regulated body weight and serum lipid levels in MI/R+hyperlipidemia rats

In comparison with MI/R group, MI/R+hyperlipidemia group demonstrated significantly higher body weight (P < 0.01). HSYA lowered the body weight of hyperlipidemic rats (shown in Supplementary Fig. 1).

**Figure 2:**
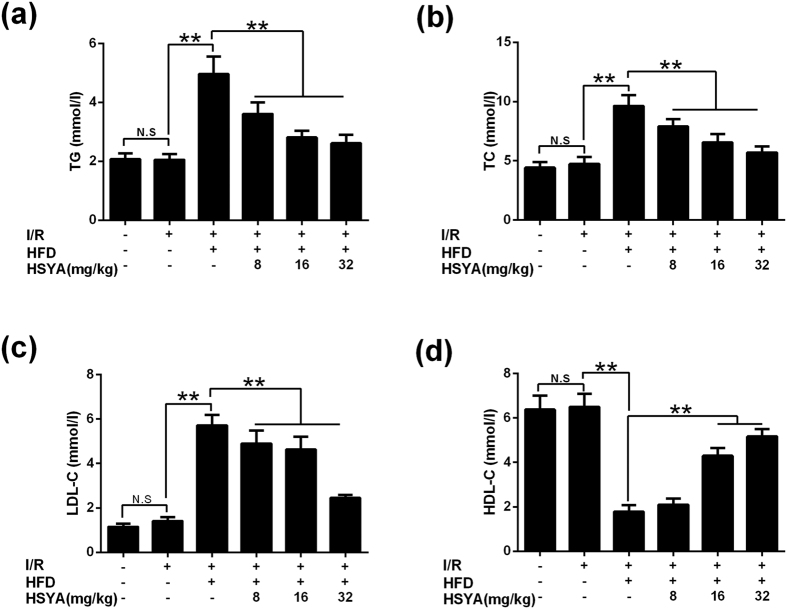
Effects of HSYA on TG, TC, LDL-C and HDL-C levels in response to MI/R+hyperlipidemia injury. (**a**) HSYA decreased TG level of MI/R+hyperlipidemia group (n = 8). (**b**) HSYA suppressed TC level of MI/R+hyperlipidemia group (n = 8). (**c**) HSYA down-regulated LDL-C concentration of MI/R+hyperlipidemia group (n = 8). (**d**) HSYA increased HDL-C level of MI/R+hyperlipidemia group (n = 8). Data were shown as mean ± S.D. ^**^P < 0.01; N.S, no significance.

Compared with sham group, MI/R did not affect TG, TC, LDL-C and HDL-C levels significantly. Rats of MI/R+hyperlipidemia group showed significantly higher TG, TC and LDL-C levels than myocardial I/R group (P < 0.01). All HSYA-treatment groups decreased TG, TC and LDL-C levels dose-dependently. HSYA (16 mg/kg and 32 mg/kg) decreased TG, TC and LDL-C levels significantly (P < 0.01), and increased HDL-C level significantly (P < 0.01) (shown in Fig. 2).

### HSYA alleviated myocardial injury and inflammation in MI/R+hyperlipidemia rats

**Figure 3:**
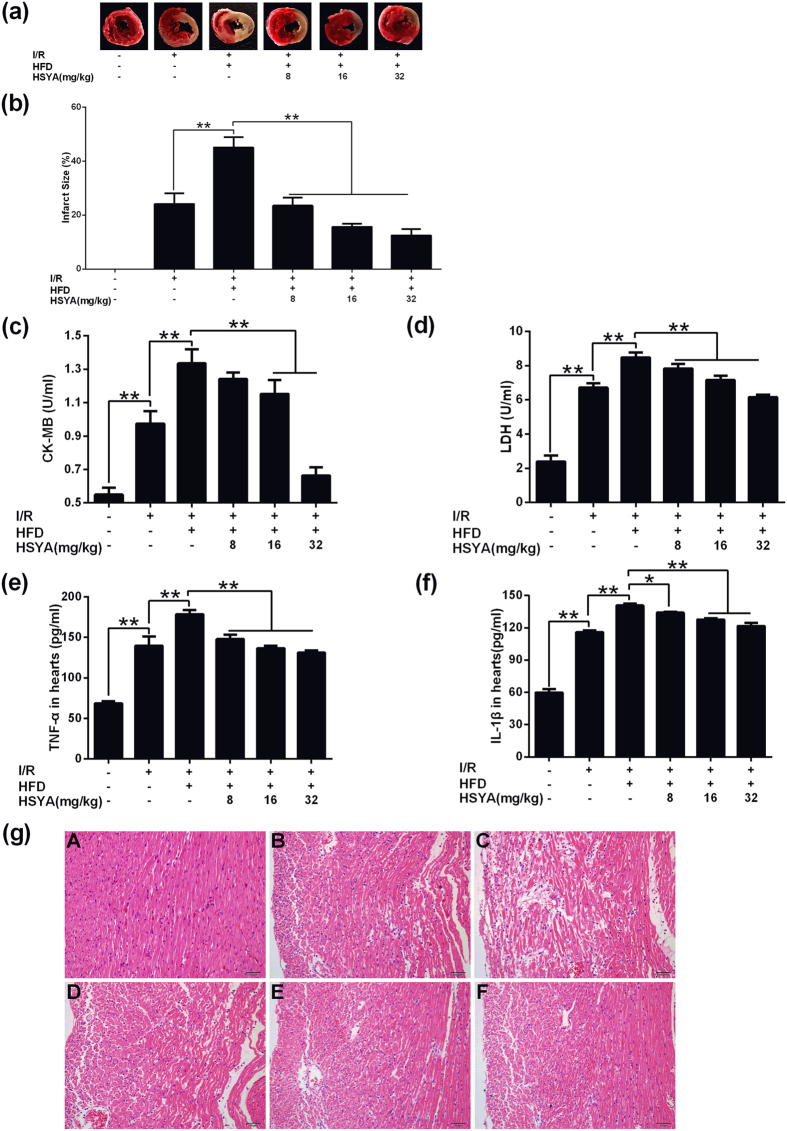
Effects of HSYA on rat heart infarct size, myocardial damage extent, inflammatory cytokine concentration, and histological features of rat cardiac tissues in response to MI/R+hyperlipidemia injury. (**a**) Representative images of rat heart slices in different group. (**b**) Quantification of rat heart infarct size in different group (n = 8). (**c**) HSYA suppressed the up-regulation of CK-MB level of MI/R+hyperlipidemia group (n = 8). (**d**) HSYA decreased LDH activity of MI/R+hyperlipidemia group (n = 8). (**e**) HSYA down-regulated the over secretion of TNF-α in rat hearts (n = 3). (**f**) HSYA decreased IL-1β expression in rat hearts. (**g**) Histological analysis representative pictures (200×) of cardiac tissues in sham (A), MI/R (B), MI/R+hyperlipidemia (C), MI/R+hyperlipidemia + HSYA 8 mg/kg (D), MI/R+hyperlipidemia + HSYA 16 mg/kg (E), MI/R+HSYA+ hyperlipidemia 32 mg/kg (F) group; n = 8. Scale bar = 50 μm. Data were shown as mean ± S.D.; ^*^P < 0.05; ^**^P < 0.01.

Firstly, we determined the rat myocardial infarct size of different groups by TTC staining. MI/R resulted in a clearly distinguishable infarct zone, as shown in Fig. 3a. MI/R+hyperlipidemia group owned significantly higher infarct size than myocardial I/R group (P < 0.01). All HSYA treatment groups exhibited significantly lower infarct size in comparison with that of MI/R+hyperlipidemia group (P < 0.01) (shown in Fig. 3b).

The activity of LDH and CK-MB in serum was used to monitor the myocardial damage. Compared with sham group, activity of LDH and CK-MB in MI/R group was elevated significantly (P < 0.01). MI/R+hyperlipidemia group showed much higher level of LDH and CK-MB than I/R group. After the treatment of HSYA, the over-production of LDH and CK-MB in serum was suppressed. HSYA (16 mg/kg and 32 mg/kg) decreased the serum LDH and CK-MB activity of MI/R+hyperlipidemia group significantly (P < 0.01) (shown in Fig. 3c,d).

Next, we investigated the effects of HSYA on cardiac inflammatory factor concentration. In comparison with sham group, MI/R group increased TNF-α and IL-1β levels significantly in rat hearts (P < 0.01). Meanwhile, MI/R+hyperlipidemia group demonstrated significantly higher levels of TNF-α and IL-1β in rat hearts than I/R group (P < 0.01). All HSYA groups ameliorated the excessive production of TNF-α and IL-1β in rat hearts induced by MI/R superimposed on hyperlipidemia injury (shown in Fig. 3e,f).

As shown in Fig. 3g, sham group exhibited normal structure without lesions, edema or neutrophils. In MI/R group, slight necrosis, myocardial structure disorder and neutrophils infiltration were observed. MI/R+hyperlipidemia group showed more serious damage than I/R group. In MI/R+hyperlipidemia group, apparent perivascular edema and structural disarray, serious necrosis, and many infiltrating neutrophils were observed. After treatment with HSYA (8 mg/kg, 16 mg/kg and 32 mg/kg), the histological features became mild architectural damage or typical of normal cardiac structure. Of note, the numbers of infiltrated neutrophils and necrosis cells in HSYA treated groups were less compared with MI/R+hyperlipidemia group (shown in Fig. 3g).

### HSYA inhibited serum LPS, TNF-α and IL-1β concentration in MI/R+hyperlipidemia rats

**Figure 4:**
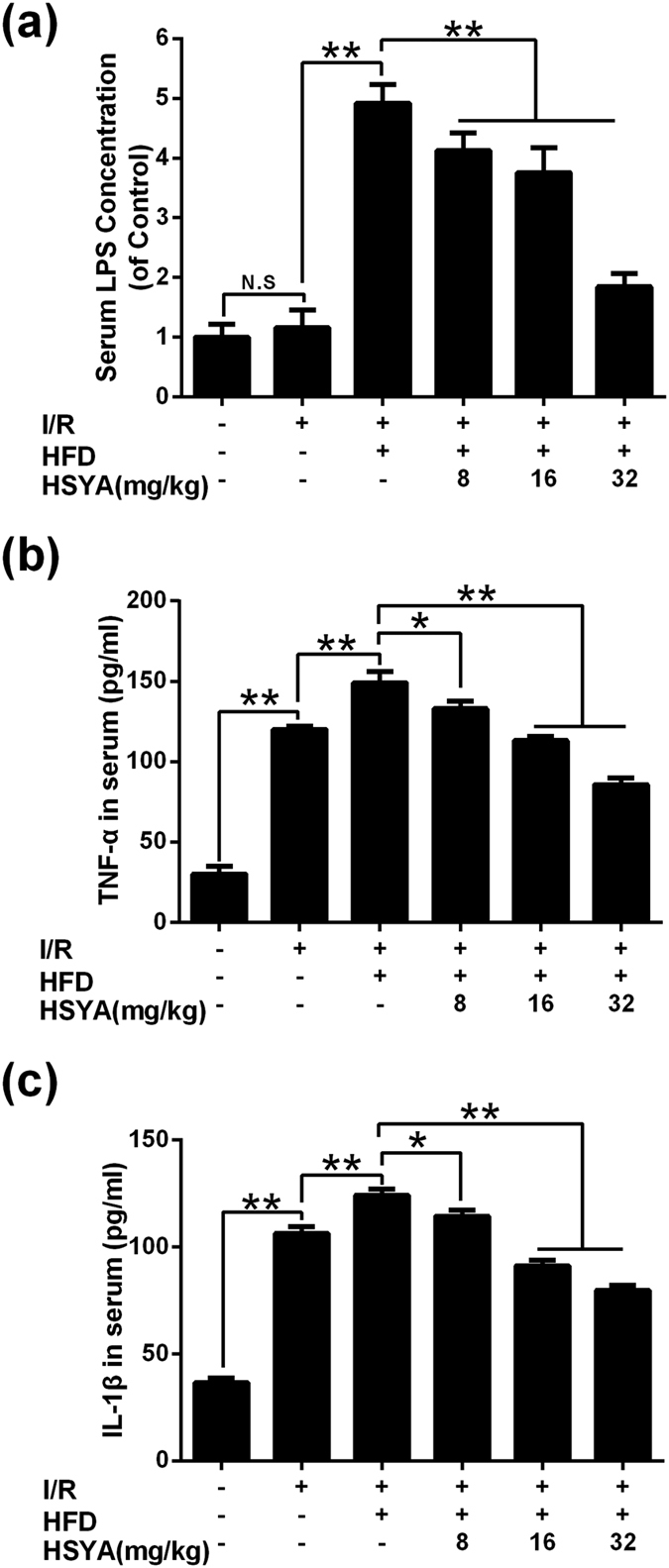
Effects of HSYA on serum LPS concentration (of sham group), TNF-α concentration and IL-1β concentration in response to MI/R+hyperlipidemia injury in rats. (**a**) HSYA lowered rat serum LPS concentration (n = 8). (**b**) HSYA suppressed the excessive release of TNF-α in rat serum (n = 3). (**c**) HSYA decreased the level of IL-1β in rat serum (n = 3). Data were shown as mean ± S.D.; ^*^P < 0.05; ^**^P < 0.01; N.S, no significance.

With the finding that HSYA protected rat hearts from injury induced by MI/R+hyperlipidemia we tested whether HSYA regulated systemic inflammatory response. Serum LPS concentration was calculated by dividing the values of different group by sham group. As shown in Fig. 4a, there was no difference between sham group and MI/R group. Compared with I/R group, MI/R+hyperlipidemia group demonstrated a significantly higher level of serum LPS (P < 0.01). All HSYA treatment groups down-regulated serum LPS concentration significantly (P < 0.01).

As shown in Fig. 4b,c, compared with sham group, MI/R group increased serum TNF-α and IL-1β levels significantly (P < 0.01). Meanwhile, MI/R+hyperlipidemia group had significantly higher levels of TNF-α and IL-1β in serum than I/R group (P < 0.01). All HSYA groups ameliorated the excessive production of TNF-α and IL-1β in serum induced by MI/R+hyperlipidemia injury.

### HSYA down-regulated over-expressed TLR4 in hearts of MI/R+hyperlipidemia rats

**Figure 5:**
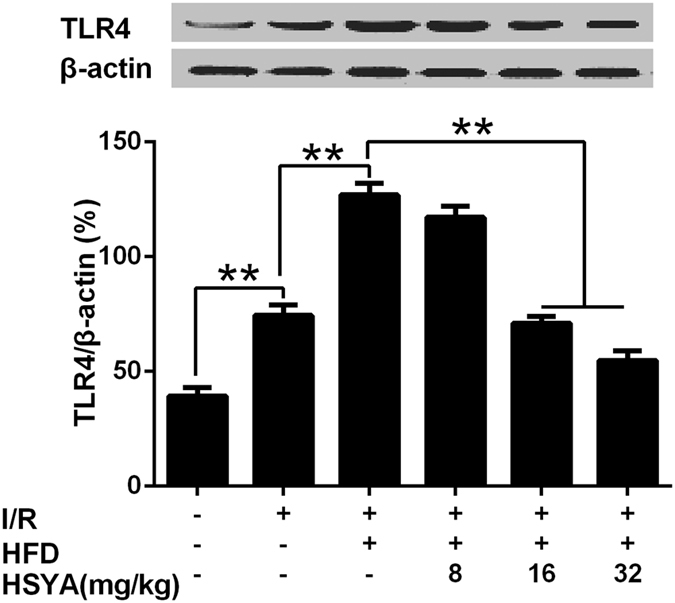
Effects of HSYA on TLR4 expression in rat hearts in response to MI/R+hyperlipidemia injury. Data were shown as mean ± S.D., n = 3. ^**^P < 0.01.

As shown in Fig. 5, TLR4 expression level was low in rat hearts of sham group. MI/R group had significantly higher level of TLR4 expression than sham group (P < 0.01). And TLR4 was up-regulated significantly in response to MI/R+hyperlipidemia than MI/R (P < 0.01). HSYA (8 mg/kg, 16 mg/kg and 32 mg/kg) suppressed the over-expression of TLR4 effectively and dose-dependently. TLR4 levels of HSYA (16 mg/kg and 32 mg/kg) groups were significantly lower than MI/R+hyperlipidemia group (P < 0.01).

### HSYA did not protect against cardiac injury and inflammatory response of hearts induced by myocardial I/R under the condition of TLR4-knockout in mice

**Figure 6:**
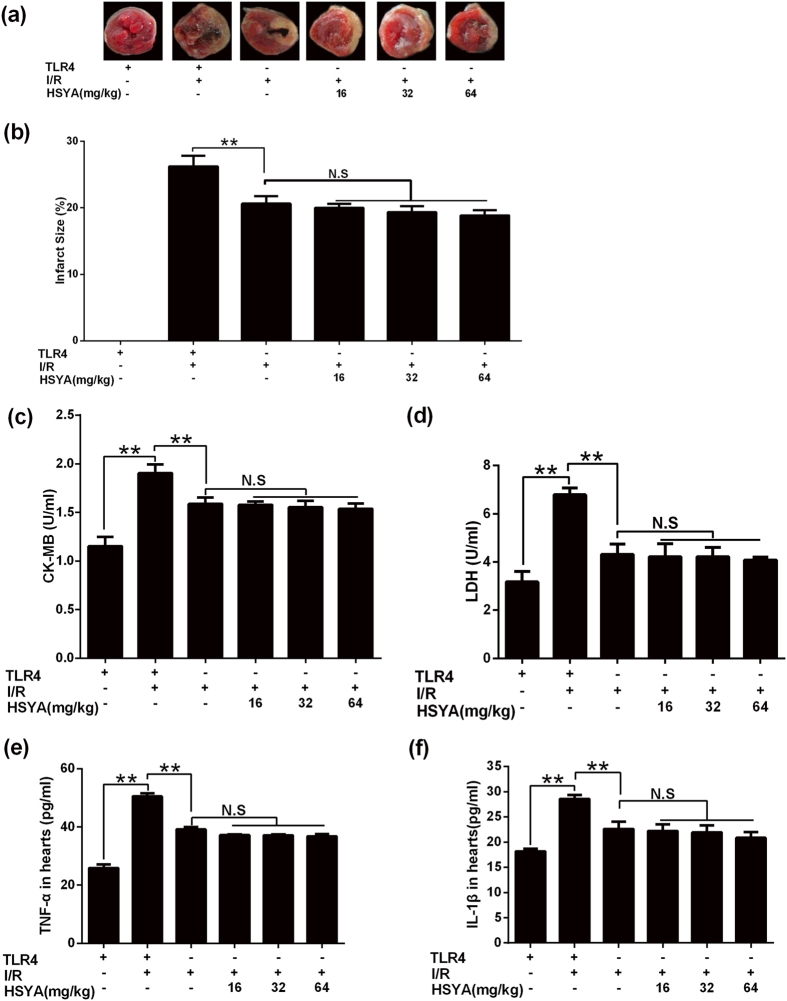
Effects of HSYA on MI/R injury in TLR4-KO mice. (**a**) Representative images of mice heart slices in different group. (**b**) Quantification of mice heart infarct size in different group (n = 6). (**c**) HSYA did not decrease serum CK-MB activity of TLR4-KO mice significantly (n = 6). (**d**) HSYA had little influence on serum LDH of TLR4-KO mice (n = 6). (**e**) HSYA down-regulated cardiac TNF-α level of TLR4-KO mice without significance (n = 3). (**f**) HSYA had little influence on cardiac IL-1β level of TLR4-KO mice (n = 3). Data were shown as mean ± S.D.; ^**^P < 0.01; N.S, no significance.

To further test whether HSYA alleviated the myocardial injury via TLR4, TLR4-KO mice subjected to MI/R were used by us. WT myocardial I/R group owned a very high infarct size; TLR4-knockout mice exhibited a significantly lower infarct size than WT mice in response to myocardial I/R insult (P < 0.01). HSYA has little influence on infarct size after TLR4 was knockout (shown in Fig. 6a,b).

Compared with WT, WT MI/R group had a significantly higher level of serum CK-MB and LDH (P < 0.01). Serum CK-MB and LDH amount in TLR4-KO MI/R group was significantly lower than WT MI/R group (P < 0.01). The activity of serum CK-MB and LDH was decreased a bit by HSYA treatment (shown in Fig. 6c,d).

WT mice had a significantly up-regulation of cardiac TNF-α and IL-1β in response to MI/R (P < 0.01). TLR4-KO MI/R group demonstrated significantly lower cardiac TNF-α and IL-1β amount in comparison to WT MI/R group (P < 0.01). The amount of cardiac TNF-α and IL-1β in HSYA groups was a little lower than TLR4-KO MI/R group (shown in Fig. 6e,f).

### HSYA increased cell viability and inhibited inflammatory response in H/R + LPS injured NRVMs

**Figure 7:**
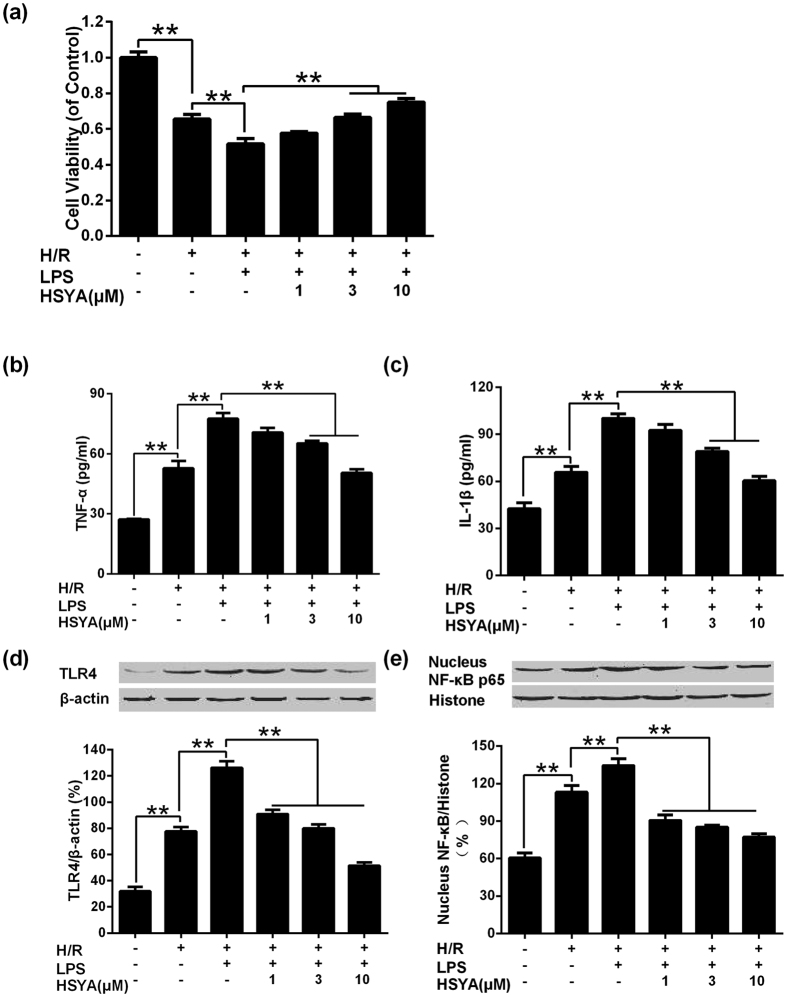
Effects of HSYA on cell viability, inflammatory cytokine concentration, TLR4 expression and nucleus NF-κB expression after LPS stimulation superimposed on H/R injury in NRVMs. (**a**) HSYA improved cell viability of NRVMs in response to H/R + LPS insult (n = 3). (**b**) HSYA suppressed the over-expression of TNF-α stimulated by H/R and LPS (n = 3). (**c**) HSYA decreased IL-1β of H/R + LPS group (n = 3). (**d**) HSYA decreased the expression of TLR4 (n = 3). (**e**) HSYA down-regulated the expression of nucleus NF-κB (n = 3). Data were shown as mean ± S.D.; ^**^P < 0.01.

In order to examine the effects of HSYA on cardiomyocytes suffered from MI/R+hyperlipidemia, NRVMs were cultured *in vitro*. Cell viability was calculated by dividing the OD values of model, HSYA group by those in the sham control group. After H/R, the viability of NRVMs was decreased significantly in comparison with sham group (P < 0.01). LPS exaggerated the injury induced by H/R markedly. Treatment with HSYA (1 μM, 3 μM and 10 μM) up-regulated the cell viability in a concentration-dependent manner. HSYA (3 μM and 10 μM) significantly ameliorated the injury induced by H/R superimposed on LPS insult (P < 0.01) (shown in Fig. 7a).

Cell culture supernatant of different groups was collected for ELISA analysis. Compared with sham group, H/R group exhibited markedly higher levels of TNF-α and IL-1β (P < 0.01). H/R + LPS group increased TNF-α and IL-1β amount significantly than those of I/R group (P < 0.01). All HSYA groups down-regulated the excessive amount of TNF-α and IL-1β and HSYA (3 μM and 10 μM) group significantly suppressed the over-production of TNF-α and IL-1β induced by H/R superimposed on LPS insult (P < 0.01) (shown in Fig. 7b,c).

TLR4 and nucleus NF-κB expression amount in NRVMs under different conditions was determined by western blot. H/R group showed significant elevation of TLR4 and nucleus NF-κB than sham group (P < 0.01); H/R + LPS group demonstrated significantly higher levels of TLR4 and nucleus NF-κB in comparison with H/R group (P < 0.01). All HSYA treatment groups decreased the over-expression of TLR4 and nucleus NF-κB induced by H/R superimposed on LPS incubation effectively and concentration-dependently (shown in Fig. 7d,e).

## Discussion

This is the first study carried out to declare whether HSYA provides protection against MI/R injury superimposed on hyperlipidemia. The results showed that HSYA inhibited hyperlipidemia induced excessive LPS release, suppressed TLR4, depressed inflammatory response, and alleviated MI/R injury *in vivo*. In NRVMs, HSYA decreased LPS + H/R induced inflammatory cytokine secretion, and down-regulated TLR4 and NF-κB over-expression.

Myocardial infarction is a common disease with serious consequences in mortality, morbidity, and cost to the society; besides, hyperlipidemic patients are prone to be complicated with myocardial ischemia^17^. However, it is still poorly understood that how hyperlipidemia augmented myocardial infarction. Hyperlipidemia could be classified as either familial hyperlipidemia or acquired hyperlipidemia. The latter type of hyperlipidemia is more common than the former one^18^,^19^. With the increase in incidence of diet-induced hyperlipidemia, this study selected high-fat diet-induced hyperlipidemia combined with MI/R injury as the model.

In this study, hyperlipidemia rats were further stimulated by MI/R injury to elucidate the effects of hyperlipidemia on MI/R. Our results showed that hyperlipidemia enhanced the injury induced by myocardial I/R in rats. MI/R+hyperlipidemia group demonstrated significantly larger infarct size and worse cardiac damage (elevation of serum CK-MB and LDH activity) than I/R group. Meanwhile, hyperlipidemia up-regulated the expression level of cardiac TNF-α and IL-1β. The histological structures of I/R+hyperlipidemia group were much more serious than I/R group. After rats were fed with high-fat diet for 8 weeks, serum TC, TG and LDL-C levels were significantly higher than normal diet-fed rats, which demonstrated that high-fat diet evoked hyperlipidemia successfully. Meanwhile, serum LPS level of hyperlipidemia rats group was significantly higher than that of normal diet-fed group, which was in line with previous studies^20^. LPS is an activator of TLR4, which is a transmembrane receptor protein. TLR4, a member of the pattern-recognition receptors family, constitutes a key node in the induction and regulation of inflammatory response^11^. TLR4 is expressed in the heart and up-regulated during myocardial I/R injury. Stimulation of TLR4 leads to the activation of intracellular signalling pathways, which, via activation of NF-κB, ultimately results in the production of pro-inflammatory cytokines, such as TNF-α and IL-1β^21^,^22^,^23^. We speculated that hyperlipidemia worsened the outcomes of rat MI/R injury via the increase of serum LPS, which augmented inflammatory response induced by I/R. To test whether hyperlipidemia sharpened the inflammatory response induced by myocardial I/R through the up-regulation of the secretion of pro-inflammatory cytokines, we analyzed the expression of pro-inflammatory cytokines TNF-α and IL-1β in serum. The results showed that hyperlipidemia stimulated the excessive expression of TNF-α and IL-1β in serum of myocardial I/R group. We also found that hyperlipidemia+MI/R group owned significantly higher TLR4 level in hearts than MI/R group.

HSYA, a monomer compound extracted from the flower of the safflower plant (*Carthamus tinctorius* L.), was proved to protect H9c2 cardiomyocytes against H/R-induced apoptosis through up-regulation of heme oxygenase-1^24^. However, there are few studies about the effect of HSYA on myocardial ischemia complicated with hyperlipidemia.

We then examined the efficacy of HSYA on high-fat diet induced hyperlipidemia rats combined with myocardial I/R injury. The results showed that HSYA reduced body weight of hyperlipidemic rats, regulated serum lipid profiles, and then suppressed serum LPS concentration, which led to the decrease of peripheral inflammatory response. HSYA also suppressed the over-expression of TLR4 in hearts induced by I/R combined with hyperlipidemia. The suppression of excessive inflammation and TLR4 over-expression by HSYA led to the decrease of the infarct size and the alleviation of cardiac damage. HSYA down-regulated the activity of serum CK-MB and LDH of MI/R+hyperlipidemia group. Meanwhile, HSYA alleviated the pathological features induced by MI/R+hyperlipidemia. From the above data, we inferred that TLR4 might be the target of HSYA in the pathological process of hyperlipidemia+MI/R.

In order to test how HSYA ameliorated cardiac injury induced by hyperlipidemia+MI/R, TLR4-KO mice were applied. The above experiments showed that hyperlipidemia augmented MI/R injury through increasing serum LPS level. It was reported that inflammatory cells of TLR4-deficient mouse model did not produce any detectable levels of proinflammatory cytokines in response to LPS^25^. Therefore, we evaluated the effects of HSYA on MI/R in TLR4-KO mice. A recent experimental study has reported that TLR4 deficiency reduces inflammatory pathways linked to the expansion of myocardial salvage in myocardial ischemia-reperfusion^26^,^27^. This study tested that TLR4-KO mice owned significantly lower infarct size, CK-MB activity, LDH activity TNF-α level, and IL-1β level than WT mice in response to MI/R. Our results also showed that HSYA failed to ameliorate cardiac injury and inflammatory response under the condition of TLR4-deficiency, which proved that HSYA restored cardiac injury induced by hyperlipidemia+MI/R through suppressing TLR4.

To further explore the in-depth mechanism of the protective effect of HSYA on cardiac cells in the model of myocardial I/R superimposed on hyperlipidemia, H/R NRVMs subjected to LPS were applied in this study. It was reported that taking high-fat diet chronically increased plasma LPS concentration^28^. Our *in vivo* study also revealed that 8-week high-fat diet induced significant increase of serum LPS. Therefore, LPS was applied *in vitro* to mimic high-fat diet induced hyperlipidemia. H/R NRVMs possessed markedly higher viability than those suffered from H/R combined with LPS insult. HSYA improved the cell viability of H/R + LPS group to a great extent. In response to MI, cardiomyocytes express proinflammatory cytokines, which are able to initiate a local inflammatory response^29^. Inflammatory responses following ischemia are modulated via TLR4^7^,^26^. In this study, H/R + LPS NRVMs over-expressed TLR4 and nucleus NF-κB. Activation of TLR4 induces the nuclear factor-κB (NF-κB) dependent expression of proinflammatory cytokines, such as tumor necrosis factor α (TNF- α) and interleukin 1β (IL-1β)^9^. NF-κB, a transcription factor, plays a role in numerous pathological states such as myocardial ischemia–reperfusion. Dissociation and degradation of IκB induce the translocation of NF-κB from the cytosol to the nucleus and facilitate the transcription of its responsive genes, including inflammatory cytokines and adhesion moleculars. Moss *et al*. have previously demonstrated that inhibition of the NF-κB translocation during reperfusion can attenuate infarct size and maintain cardiac function^30^. Pro-inflammatory cytokines, such as TNF-α and IL-1β are not constitutively expressed in the normal heart^31^. The up-regulation and production of these cytokines induced by nucleus NF-κB accumulation represent an intrinsic or an innate stress response against myocardial injury^32^. In this study, the levels of TNF-α and IL-1β in H/R + LPS group were significantly higher than H/R group, which implied that cardiomyocytes of H/R + LPS group suffered more severe inflammatory response than H/R group. HSYA alleviated the over-expression of TLR4 and the accumulation of nucleus NF-κB, then suppressed the up-regulation of TNF-α and IL-1β.

In our study, serum LPS level and myocardial TLR4 expression level were increased in response to MI/R+hyperlipidemia. Traditionally, TLRs serve as key sensors of PAMPs, such as bacterial LPS, lipopeptides, and flagellins, which exist in microbial cells but not host cells. TLRs have therefore been considered to play a central role in the discrimination between “self” and “non-self”; however, more recently, diverse molecules originated from host cells may also serve as endogenous ligands of TLR4, which serve as mediators of inflammation. It was proposed that endogenous TLR4 ligands may stimulate TLR4 signaling directly^33^,^34^,^35^. Potential endogenous TLR4 ligands maybe also involved in the pathological process of MI/R+hyperlipidemia, which will be investigated in future study.

**Figure 8:**
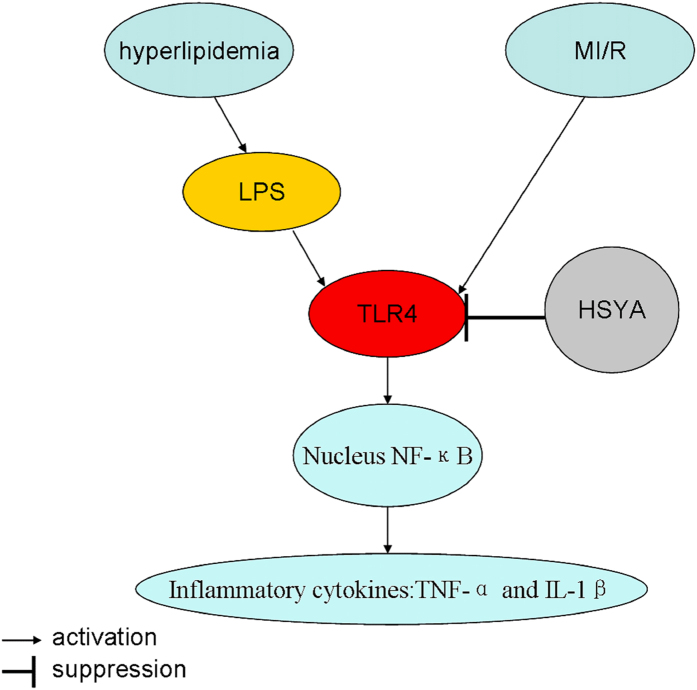
Schematic summary showing that how HSYA alleviated hyperlipidemia superimposed on MI/R injury.

Taken together, our results revealed that hyperlipidemia augmented MI/R injury through LPS combining with TLR4. High-fat diet-induced hyperlipidemia exaggerated the outcomes of myocardial I/R in rats through inflammatory response and HSYA could relieve the cardiac injury caused by MI/R+hyperlipidemia through suppressing TLR4 *in vivo* and *in vitro* (Fig. 8).

## Methods

### Reagents

HSYA was obtained from Shanxi Huahuikaide Pharmaceutical Co., Ltd (Jinzhong, China) as a yellow amorphous powder. Its purity (>98%) was determined by HPLC. It is soluble in water and has a molecular formula of C_27_H_32_O_16_. 2,3,5-Triphenyltetrazolium chloride (TTC) and 3- (4,5-dimethylthiazol-2-yl) -2,5-diphenyltetrazolium bromide (MTT) were purchased from Sigma. TNF-α and IL-1β enzyme-linked immunosorbent assay (ELISA) kits were purchased from Nanjing Jiancheng Bioengineering Institute. All antibodies were obtained from Santa Cruz Biotechnology, Inc. (CA, USA). All other reagents were of analytical grade and commercially available.

### Animals and protocols

Male Wistar rats (250–280 g) were provided by Zhejiang Laboratory Animals Center (Hangzhou, China). Eight to 12-week-old adult male WT (C57BL) and TLR4-knockout (TLR4-KO) mice (20–25 g) were purchased from Model Animal Research Center of Nanjing University (Nanjing, China). Experiments were approved by Ethics Committee for Animal Experimentation of China Pharmaceutical University (Nanjing, China) and were carried out in accordance with Guidelines for the Care and Use of Laboratory Animals of China Pharmaceutical University.

Hyperlipidemia combined with MI/R model: rats were fed with a standard (normal diet) or HFD (supplemented with 1.2% cholesterol, 0.2% methylthiouracilum, 15% lard, 15% sucrose and 0.5% sodium cholate). Plasma lipid levels were measured 4 weeks later, and animals with hyperlipidemia were divided into different groups randomly to receive the HFD and drug treatment for another 4 weeks. The rats of different groups received isovolumetric saline or HSYA intraperitoneally (i.p.), once per day. For the MI/R experiment, rats were anesthetized with chloral hydrate (300 mg/kg i.p.) and ventilated by a respirator (HX-100E, Taimeng Co. Ltd., China) with a tidal volume of 10 ml/kg and a respiratory rate of 80 cycles per minute. A left thoracotomy was performed and the pericardium was opened to expose the heart. A 5-0 silk suture was placed around the left arterial descending (LAD) coronary artery appendage for 2–3 mm through a small polytetra fluoroethylene tube, which formed a snare. Myocardial ischemia was induced by one stage occlusion of the LAD for 30 min. Then the myocardium was reperfused by releasing the snare gently for a period of 2 hours. The sham-operated rats underwent the same thoracotomy without LAD ligation and release. Coronary occlusion was confirmed by dramatic change in color (pale) of the area ligated^36^. The body weight was recorded before animals were sacrificed. The experimental groups were shown in Table 1.

MI/R model: mice of different groups received isovolumetric saline or HSYA i.p., once per day, for 7 days before MI/R was conducted. For the MI/R experiment, mice were anesthetized with chloral hydrate (300 mg/kg i.p.) and ventilated by a respirator with a respiratory rate of 120 cycles per minute. A left thoracotomy was performed and the pericardium was opened to expose the heart. A 7-0 silk suture was placed around the LAD coronary artery appendage for 1–2 mm through a small polytetra fluoroethylene tube, which formed a snare. Myocardial ischemia was induced by occlusion of the LAD for 30 min and the reperfusion is last for 2 hours. The sham-operated mice underwent the same thoracotomy without LAD ligation and release. The experimental groups were shown in Table 2.

### Measurement of serum lipid profiles

Before rats were sacrificed, blood samples for ELISA analysis were collected. The blood was centrifuged at 4000 rpm for 5 min to remove cells. Serum total cholesterol (TC), triglycerides (TG), high-density lipoprotein cholesterol (HDL-C) and low lipoprotein cholesterol (LDL-C) levels were determined by commercially available kits according to the protocol.

### Measurement of serum LPS concentration

Serum LPS concentration was determined by kinetic-QCL™ LAL kit from Lonza (Walkersville, MD, USA) using a micro-plate reader. Before rats were sacrificed, blood samples for analysis were collected. The blood was centrifuged at 4000 rpm for 5 min to remove cells. Serum samples used for LPS determination were stored in LPS-free glass tubes to prevent loss of endotoxin to plastic tube walls. Serum was diluted 10-fold and heated to 75 °C for 5 min before LPS measurement.

### Determination of CK-MB and LDH levels in serum

Myocardial cellular damage was evaluated by measuring serum CK-MB and LDH levels 2 hours after reperfusion. Serum was collected as the above mentioned method, and serum CK-MB and LDH of rats and mice were quantified using a commercial kit according to the manufacturer’s instructions.

### ELISA analysis for TNF-α and IL-1β concentration in serum and hearts

Serum was collected for TNF-α and IL-1β level which were determined by corresponding ELISA kits. After animals were sacrificed, hearts were collected and homogenized, and the homogenate was diluted in saline for ELISA analysis. Experiments were conducted according to the protocol in triplicate.

### Analysis of myocardial infarction

The left ventricle area was sectioned into five slices from the apex to the base and incubated in 2% TTC in pH 7.4 buffer for 15 min at 37 °C. TTC stains viable tissue dark red while the infarct portion remains grayish-white. Slices were photographed with a digital camera. The infarcted area in each section was measured using an Image-Pro Plus analysis system. The total infarct volume was determined by integrating the areas from all sections, and the results were expressed as a percentage of the total volume of the heart.

### Histopathological examination of cardiac tissues

Hearts were fixed in 10% formalin and embedded in paraffin. The paraffin-embedded tissues were sectioned and stained with hematoxylin–eosin (H&E) and analyzed by light microscopy. The histological sections were examined by an observer blinded to the treatment regimen.

### Western blot analysis of TLR4 in hearts

Cytoplasmic extracts of infracted hearts were prepared as described previously^37^ and the quantity of total protein was assessed by BCA assay. Then 50 μg of protein was separated by SDS-PAGE gel electrophoresis and polyvinylidene fluoride membrane. Membrane was blocked with 5% skim milk in 10 mM Tris-HCl containing 150 mM sodium chloride and 0.5% Tween 20 (TBS-T). After being washed with TBS-T, the membrane was incubated with primary antibody respectively. Membranes were detected using horseradish peroxidase-conjugated antibody. The blots were visualized by enhanced chemiluminescence detection kit. The experiment was repeated in triplicate and the bands were quantified with imageJ analysis software.

### Isolation and culture of neonatal rat ventricular myocytes (NRVMs)

NRVMs were isolated from 2-day-old Wistar rats by enzymatic digestion with 0.05% trypsin and 0.04% collagenase, as described previously^38^. Cardiac fibroblasts were removed, and NRVMs were plated onto 35-mm dishes at a density of 1 × 10^6^ cells/ml in Dulbecco’s modified Eagle’s medium (DMEM) containing 10% fetal bovine serum (FBS, Hyclone, Australian), 100 units/ml penicillin/streptomycin, and 0.1 mmol/l 5-bromo-2-deoxyuridine to inhibit fibroblast proliferation at 37 °C with a humidified 95% to 5% (v/v) mixture of air and CO_2_.

NRVMs were preincubated with PBS and HSYA (1 μM, 3 μM, and 10 μM) respectively in DMEM for 24 h and washed twice with PBS. Except sham group, the cells were incubated with DMEM (low glucose) containing LPS (1 μg/ml) or isovolumetric PBS in an oxygen-free incubator (95% N_2_/5% CO_2_) for 2 h (hypoxia+LPS/hypoxia). After that, the cells were incubated with DMEM in a humidified 95% air and 5% CO_2_ at 37 °C for 2 h (reoxygenation). Hypoxia/reoxygenation (H/R) model was employed *in vitro* to mimic I/R injury *in vivo*.

### Measurement of cell viability

Cell viability was determined using the MTT assay. After cells were treated under different conditions, the medium was replaced with an equal volume of fresh medium containing 5 mg/ml MTT and incubated for 4 h at 37 °C. Then MTT was removed, and cells were lysed with dimethyl sulfoxide (DMSO) with stirring for 15 min on a microtiter plate shaker. The cells’ viability was estimated according to the optical density (OD) values determined by a microplate reader at an absorption wave length of 570 nm.

### ELISA analysis for TNF-α and IL-1β concentration in cell culture supernatant

Inflammatory cytokine levels of TNF-α and IL-1β in culture supernatant were measured after cells were treated under different conditions. After the medium was centrifuged at 1500 rpm for 5 min, the supernatant was collected for the determination of TNF-α and IL-1β by ELISA kits according to the manufacturer’s instructions.

### Western blot analysis of TLR4 and nucleus NF-κB

Cytoplasmic and nuclear extracts were prepared as described previously^37^ and the quantity of total protein was assessed by BCA assay. Western blot analysis procedures were as above.

### Statistical analysis

The experimental results are expressed as the mean ± S.D. Data was analyzed by SPSS 13.0 and P-value of less than 0.05 was considered statistically significant. Studies were assessed by the method of one-way variance (ANOVA) followed by Bonferroni post-hoc test to analyze the difference. All statistical figures were performed with Graph Pad Prism Version 6.0.

## Additional Information

**How to cite this article**: Han, D. *et al*. Hydroxysafflor yellow A alleviates myocardial ischemia/reperfusion in hyperlipidemic animals through the suppression of TLR4 signaling. *Sci. Rep.* **6**, 35319; doi: 10.1038/srep35319 (2016).

## Supplementary Material

Supplementary Information

## Figures and Tables

**Figure 1:**
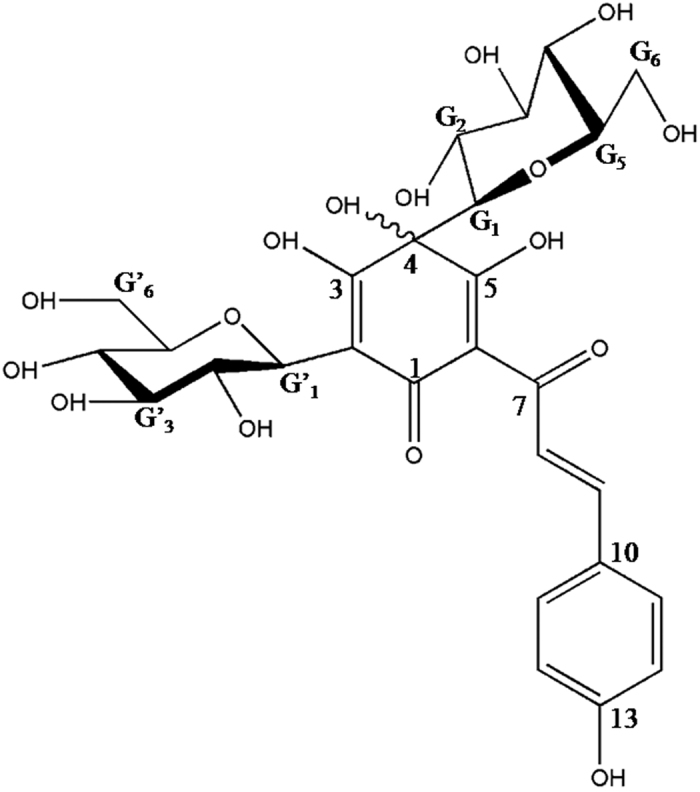
The chemical structure of hydroxysafflor yellow A.

**Table 1:** Experimental groups and protocols of rats.

Group	Drug dose	Diet pattern for 8 weeks	Surgery	n
Sham	—	Standard diet	—	8
MI/R	—	Standard diet	MI/R	8
MI/R+hyperlipidemia	—	High-fat diet	MI/R	8
MI/R+hyperlipidemia+HSYA	8 mg/kg	High-fat diet	MI/R	8
MI/R+hyperlipidemia+HSYA	16 mg/kg	High-fat diet	MI/R	8
MI/R+hyperlipidemia+HSYA	32 mg/kg	High-fat diet	MI/R	8

**Table 2:** Experimental groups and protocols of mice.

Group	Drug dose	Surgery	n
WT	—	—	6
WT MI/R	—	MI/R	6
TLR4-KO MI/R	—	MI/R	6
TLR4-KO MI/R+HSYA	16 mg/kg	MI/R	6
TLR4-KO MI/R+HSYA	32 mg/kg	MI/R	6
TLR4-KO MI/R+HSYA	64 mg/kg	MI/R	6
