# Supplementary material for: Hydroxysafflor yellow A alleviates myocardial ischemia/reperfusion in hyperlipidemic animals through the suppression of TLR4 signaling
Source: Sci Rep. 2016 Oct 12;6:35319. doi: 10.1038/srep35319 (PMC5059673; doi:10.1038/srep35319)
Supplement: Supplementary Information [file srep35319-s1.doc]

**Supplementary Information** for manuscript “Hydroxysafflor yellow A alleviates myocardial ischemia/reperfusion in hyperlipidemic animals through the suppression of TLR4 signaling”

Dan Han, Jie Wei, Rui Zhang, Wenhuan Ma, Chen Shen, Yidong Feng, Nian Xia, Dan Xu, Dongcheng Cai, Yunman Li^**^, Weirong Fang^*^


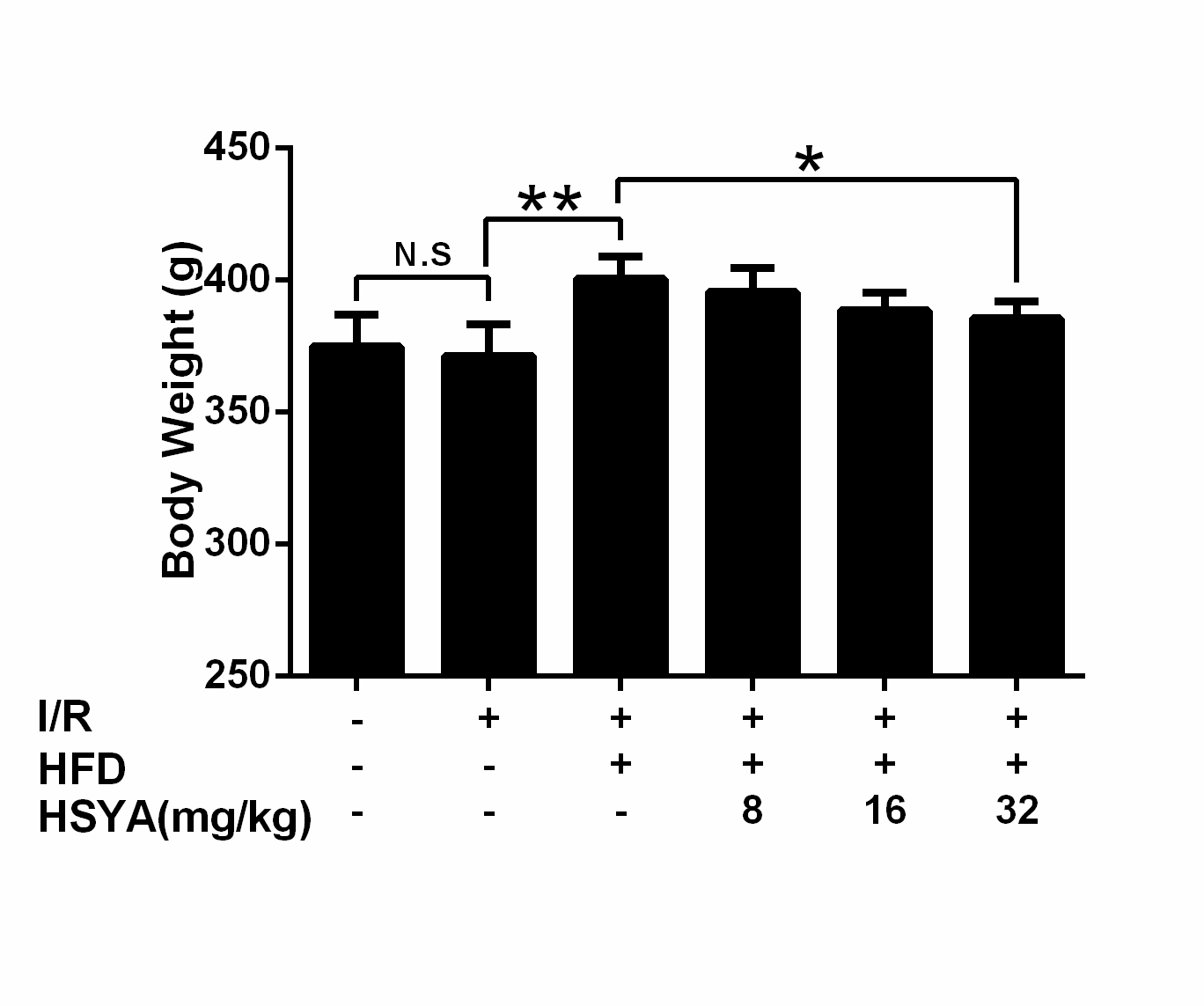


**Supplementary Figure 1** The Effects of HSYA on rat body weight in MI/I+hyperlipidemia model. Hyperlipidemia+MI/R group exhibited significantly higher body weight than MI/R group. HSYA (32mg/kg) suppressed the increase of body weight induced by hyperlipidemia significantly. Data were shown as mean ± S.D., n=8; ^*^P < 0.05; ^**^P < 0.01; N.S, no significance.
